# Activity of anthracycline- and ifosfamide-based chemotherapy in a series of patients affected by advanced myxofibrosarcoma

**DOI:** 10.1186/s13569-017-0082-6

**Published:** 2017-08-22

**Authors:** Vittoria Colia, Marco Fiore, Salvatore Provenzano, Elena Fumagalli, Rossella Bertulli, Carlo Morosi, Angelo P. Dei Tos, Marta Barisella, Alessandro Gronchi, Paolo G. Casali, Roberta Sanfilippo

**Affiliations:** 1 Medical Oncology Unit 2, Medical Oncology Department, Fondazione IRCCS Istituto Nazionale Tumori, 20133 Milan, Italy; 2 Department of Surgery, Fondazione IRCCS Istituto Nazionale Tumori, Milan, Italy; 3 Department of Radiology, Fondazione IRCCS Istituto Nazionale Tumori, Milan, Italy; 4 Department of Diagnostic Pathology, General Hospital, Treviso, Italy; 5 Department of Diagnostic Pathology and Laboratory Medicine, Fondazione IRCCS Istituto Nazionale dei Tumori, Milan, Italy; 6 Oncology and Haemato-Oncology Department, University of Milan, Milan, Italy

**Keywords:** Soft tissue sarcoma, Myxofibrosarcoma, Chemotherapy, High-dose prolonged-infusion ifosfamide

## Abstract

**Background:**

We report on the activity of anthracycline-based and high-dose prolonged-infusion ifosfamide chemotherapy in a retrospective series of patients affected by advanced myxofibrosarcoma treated at Istituto Nazionale Tumori in Milan, Italy, and within the Italian Rare Cancer Network (RTR).

**Methods:**

Advanced myxofibrosarcoma patients treated with anthracycline + ifosfamide and high-dose prolonged-infusion ifosfamide as a single agent from November 2001 to December 2016 were retrospectively reviewed. All pathological diagnosis were centrally reviewed by at least two expert pathologists. Response was evaluated by RECIST, and survival functions were computed.

**Results:**

Among 34 advanced myxofibrosarcoma patients, 13 were treated with front-line anthracycline + ifosfamide chemotherapy (male/female = 6/7, median age 54 years, range 33–72). Overall best response was: 4 partial responses, 3 stable diseases and 6 progressive diseases, with a median progression-free survival of 4 months. Twenty-eight patients received second/further line high-dose prolonged-infusion ifosfamide (male/female = 17/11, median age 55 years, range 27–75 years). We observed 10 partial responses, 4 stable diseases and 14 progressive diseases, with a median progression-free survival of 4 months. Median overall survival was 12 months.

**Conclusions:**

This retrospective analysis suggests that the combination of anthracyclines and ifosfamide is active in myxofibrosarcoma. In patients already treated with a combination of anthracyclines and ifosfamide, high-dose prolonged-infusion ifosfamide showed activity as well.

## Background

Myxofibrosarcoma is a rare soft tissue sarcoma (STS) with an estimated incidence <0.1/100.000/years [1]. Historically, it was first described in 1977 as a part of the spectrum of myxoid fibroblastic malignancies [2, 3]. It was finally included in the WHO classification in 2002 as a distinct entity with unique clinicopathological characteristics and cytoarchitectural features [4].

Myxofibrosarcoma is more common in elderly patients and it is mainly located to the extremities, especially lower limbs, but it can arise in the trunk and in head and neck region [5–7]. Since a presentation in the abdominal cavity or in the retroperitoneum is extremely rare, a differential diagnosis between dedifferentiated liposarcoma and myxofibrosarcoma should be always considered [8–10]. In comparison to the other soft tissue sarcoma types, myxofibrosarcoma exhibits an overall better prognosis [10–13], with an overall survival (OS) of approximatively 70% and an overall risk of metastases between 20 and 25% in high-grade variants [10–19]. However, myxofibrosarcoma tends to have a remarkable rate of local recurrences compared to other soft tissue sarcomas, reported between 20 and 75% [2, 5, 6, 10, 12, 16].

Surgery alone or in combination with radiotherapy is the main treatment for localized disease [10, 12], and medical treatment is used in the advance disease. The combination of anthracyclines and ifosfamide as front-line therapy of advanced soft tissue sarcomas is associated with response rates of 20–30% in previously untreated patients. This percentage drops to 10% in second line. However, this reflects the whole group of soft tissue sarcomas, which make up a heterogeneous disease with discrepant chemosensitivity across the histological subtypes. For example, angiosarcoma or myxoid liposarcoma have been observed to be responsive to chemotherapy, while other subgroups are poorly responsive [20]. In the subgroup of myxofibrosarcomas, the precise role of chemotherapy remains undefined and no prospective trials or retrospective analyses are available [21].

Therefore, we carried out this retrospective study on a series of patients affected by advanced myxofibrosarcoma treated with chemotherapy at the Istituto Nazionale Tumori, Milano, Italy, and within the Italian Rare Cancer Network, a collaborative network sharing clinical cases and data of rare cancers in Italy.

## Methods

Thirty-four patients affected by advanced myxofibrosarcoma, consecutively treated with chemotherapy between November 2001 and December 2016 at Fondazione IRCCS Istituto Nazionale Tumori, Milan, Italy, and within the Italian Rare Cancer Network, were retrospectively identified. We retrieved a subgroup of 13 patients treated with front-line anthracycline + ifosfamide chemotherapy and a subgroup of 28 patients treated with second/further line high-dose prolonged-infusion ifosfamide.

Pathological diagnosis was centrally reviewed by at least two expert pathologists in all the cases, following the most recent updated criteria [4].

Data regarding clinical and histopathological characteristics, staging, surgical and systemic treatment and survival were collected. The clinical records were reviewed and collected in one institutional database and a descriptive analysis was performed. Data on chemotherapy tolerability and adverse effects were recorded. Response to chemotherapy was evaluated with Response Evaluation Criteria in Solid Tumours (RECIST) applied to CT and/or MRI scans performed periodically (usually every 2–3 months) [22].

This retrospective analysis was approved by the Institutional Ethics Committee of our Institution.

### Treatment

When an anthracycline was used in combination with ifosfamide, the regimen was epirubicin 105 mg/sqm + ifosfamide 9000 mg/sqm, i.v., in 3 days every 3 weeks, in association with equidose MESNA, and antiemetics. Prophylactic granulocyte colony stimulating factors were given. High-dose ifosfamide (HDIFX) was administered as a single agent, at the daily dose of 1 g/sqm (total dose of 14 g/sqm per cycle), as a 14-day continuous infusion with equidose MESNA, through two portable infusional devices lasting 7 days each. HDIFX regimen was administered every 4 weeks (2 weeks of infusion followed by 2 weeks off).

### Statistical analysis

Progression-free Survival (PFS) and Overall Survival (OS) were estimated with Kaplan–Meier method [23]. Failure for PFS were death or progressive disease according to RECIST. Failure for OS was death due to any cause. Patients alive were censored at the time of the last contact.

## Results

### Anthracycline + ifosfamide chemotherapy

Thirteen patients affected by advanced myxofibrosarcoma were treated with front-line anthracycline + ifosfamide chemotherapy. Patient characteristics are shown in Table 1. Female patients were prevalent (female/male 7/6); median age at the time of the operation was 54 years (range 33–72 years).

**Table 1:** Patients treated with front-line anthracycline + ifosfamide chemotherapy: clinical characteristics and response evaluation

Patients ID	Gender	Age at time of diagnosis (years)	Myxofibrosarcoma grade	Site of primary tumour	Site of relapse at time of CT	Response evaluation criteria in solid tumours (RECIST) evaluation
1	M	53	2	Left thigh	Lung	PR
2	F	72	2	Right arm	Right arm	SD
3	M	71	3	Thoracic wall	Lung	PD
4	F	33	3	Abdomen	Lung and abdomen	PR
5	F	48	1	Left thigh	Left thigh	SD
6	F	64	1	Scalp	Scalp	PR
7	M	55	2	Thoracic wall	Lung	PD
8	F	44	3	Right thigh	Right thigh	PD
9	F	51	3	Left thigh	Lung	SD
10	M	64	2	Abdomen	Lung	PD
11	M	64	2	Left thigh	Lung	PD
12	F	33	3	Left thigh	Lung and abdomen	PD
13	M	48	2	Right thigh	Lung	PR

*M* male, *F* female, *CT* chemotherapy, *PR* partial response, *SD* stable disease, *PD* progressive disease

**Fig. 1:**
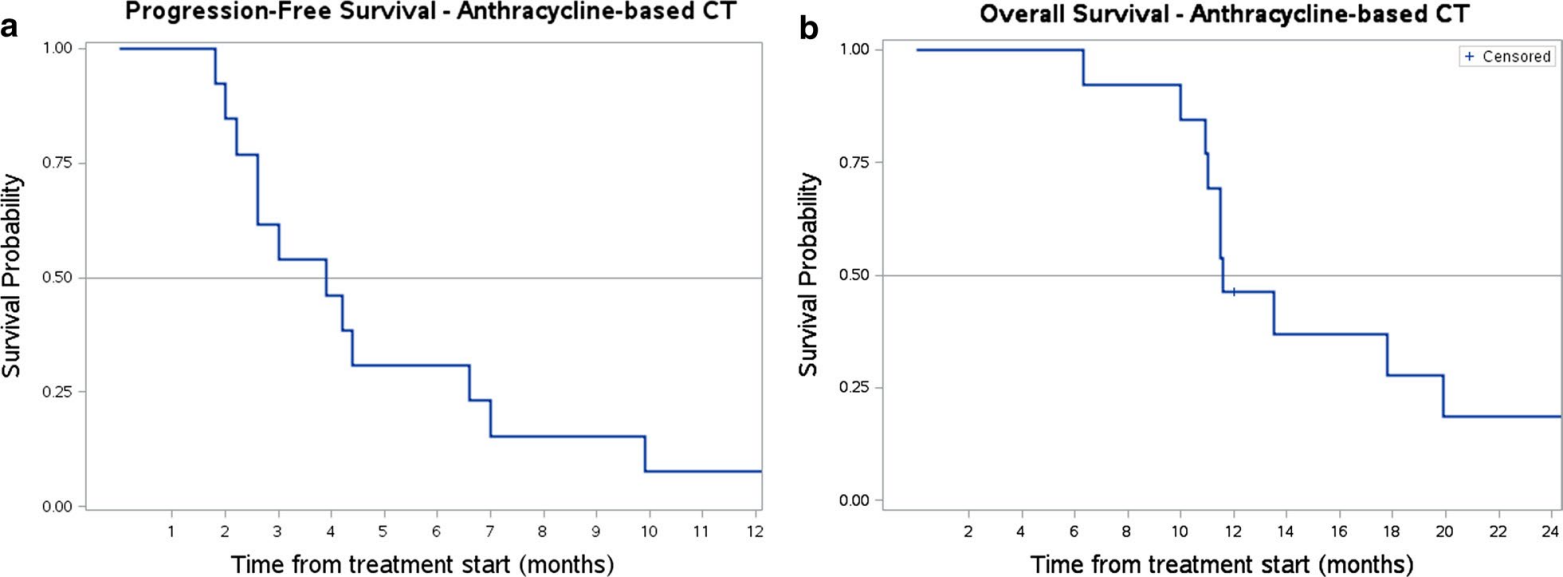
Survival functions for patients treated with front line anthracycline + ifosfamide chemotherapy (13 patients). **a** PFS (median 4 months). **b** OS (median 12 months)

All patients treated were assessable for response. Median number of chemotherapy cycles was 3 (range 2–6). The best response according to RECIST was: partial response (PR) in 4/13 cases (31%, 95% CI 0.09–0.61), stable disease (SD) in 3/13 (23%, 95% CI 0.05-0.53), progressive disease (PD) in 6/13 (46%, 95% CI 0.19–0.74) cases. Responses were confirmed at 3 months. Median PFS was 4 months, with 30% of patients progression-free at 6 months (Fig. 1a). Median OS was 12 months, with 12 patients dead and one patient alive at the time of this analysis (Fig. 1b).

Two patients interrupted their treatment for febrile neutropenia after 3 cycles of chemotherapy. No toxic deaths or any unexpected major toxicities were reported.

### High-dose ifosfamide chemotherapy

Twenty-eight patients received second/further HDIFX. Fourteen patients received epirubicin and ifosfamide in the adjuvant setting, 9/28 patients were pretreated with first line epirubicin and ifosfamide chemotherapy combination and 5/28 were pretreated with other regimens (2/5 epirubicin alone, 1/5 dacarbazine, 1/5 docetaxel, 1/5 gemcitabine). Patient characteristics are detailed in Table 2. Male patients were prevalent (male/female 17/11); median age at the time of the treatment was 55 years (range 27–75 years). Median number of HDIFX cycles was 3 (range 2–8).

**Table 2:** Patients treated with second/further-line HDIFX chemotherapy: clinical characteristics and response evaluation

Patients ID	Gender	Age at time of diagnosis (years)	Myxofibrosarcoma grade	Site of primary tumour	Site of relapse at time of CT	Response evaluation criteria in solid tumours (RECIST) evaluation
1	F	62	3	Groin	Abdomen	PD
2	F	43	3	Right arm	Lung	PD
3	M	71	2	Left thigh	Lung	PR
4	M	54	2	Left thigh	Lung	PR
5	F	72	2	Right arm	Local relapse	SD
6	M	46	3	Thoracic wall	Local relapse	RC
7	F	60	3	Right arm	Lung	PD
8	M	74	2	Left thigh	Lung	PD
9	M	57	1	Groin	Local relapse	PR
10	F	33	3	Abdomen	Lung and abdomen	PR
11	M	34	3	Right thigh	Lung	PD
12	M	71	3	Right arm	Lung	PR
13	M	70	1	sex cord	Local relapse	PR
14	F	48	1	Left thigh	Local relapse	SD
15	M	51	2	Left thigh	Lung	PD
16	F	27	2	Abdomen	Abdomen	SD
17	M	67	1	Left thigh	Local relapse	PD
18	M	56	2	Thoracic wall	Lung	PD
19	F	44	3	Right thigh	Local relapse	PD
20	M	51	3	Left leg	Lung	PR
21	M	64	2	Abdomen	Lung	PD
22	F	75	2	Right arm	Local relapse	PR
23	M	50	1	Right thigh	Local relapse	PR
24	M	65	2	Left thigh	Lung	PD
25	M	58	3	Left arm	Lung	PD
26	F	33	3	Left thigh	Lung and abdomen	PD
27	F	50	1	Thoracic wall	Lung and abdomen	SD
28	M	51	3	Left thigh	Lung	PD

*M* male, *F* female, *PR* partial response, *SD* stable disease, *PD* progressive disease

**Fig. 2:**
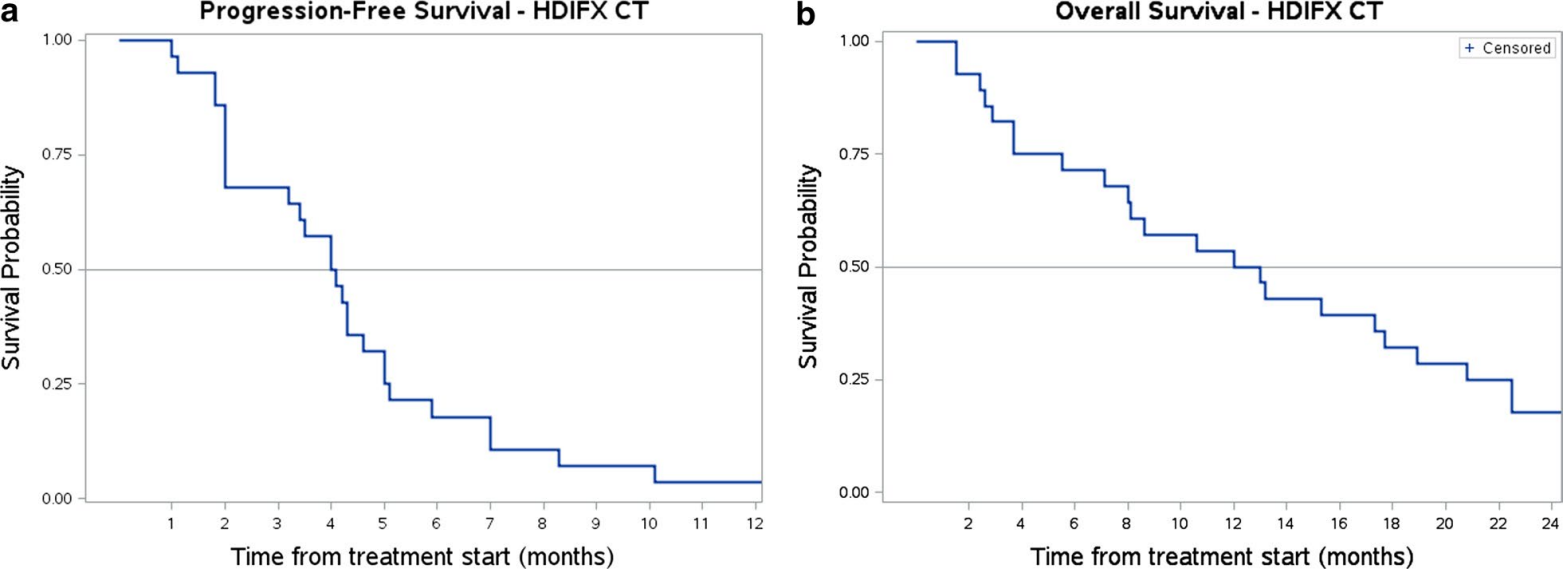
Survival functions for patients treated with continuous infusion HDIFX (28 patients). **a** PFS (median 4 months). **b** OS (median 12 months)

All patients treated with HDIFX were evaluable for response. The best response was: PR in 10/28 (36%, 95% CI 0.18–0.55), SD in 4/28 (14%, 95% CI 0.04–0.32) and PD in 14/28 (50%, 95% CI 0.30–0.69). Among patients pretreated with first line epirubicin–ifosfamide, we observed 4/9 PD (44%), 3/9 PR (33%), 2/9 SD (23%); all patients responding to second line HDIFX had had a PR to first-line epirubicin–ifosfamide. Median PFS was 4 months (Fig. 2a). Median OS was 12 months (Fig. 2b), with 27 patients dead and one patient alive at the time of this analysis.

No febrile neutropenia and renal failure were observed, as well as no toxic deaths or any other unexpected major toxicities.

**Fig. 3:**
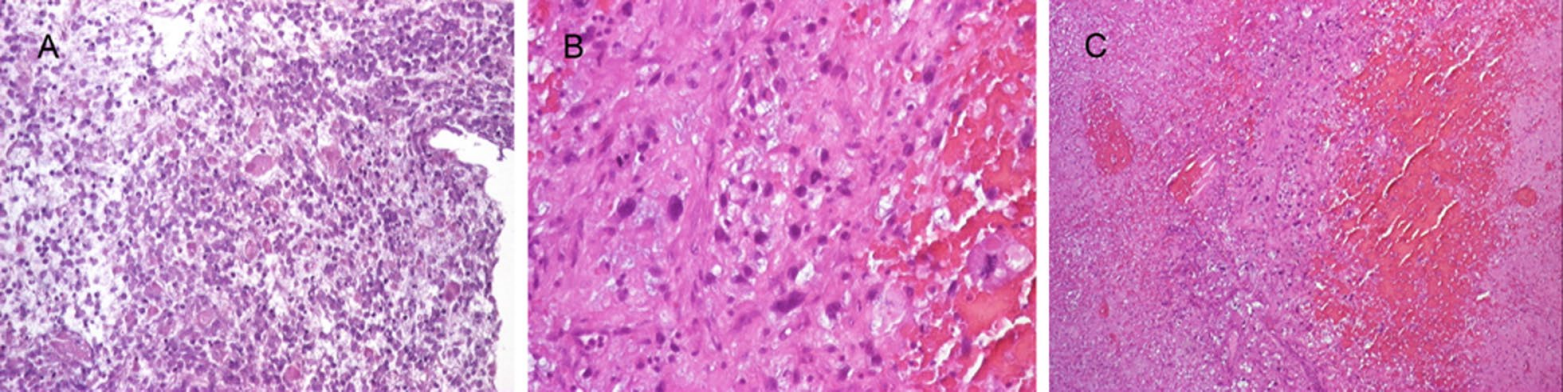
On haematoxylin and eosin staining, a pathologic partial response in a patient treated with six cycles of high-dose ifosfamide. **a** A thoracic wall metastasis from a myxofibrosarcoma arising from the thoracic wall at baseline. **b** In high power and **c** the pathologic partial response after 6 months of treatment with high-dose ifosfamide with areas of necrosis included both vascular and avascular pattern and vital areas

Figure 3 shows a pathological partial response to HDIFX.

## Discussion

In a retrospective study on a series of 34 consecutive patients with progressing advanced myxofibrosarcoma treated with chemotherapy on a 15-year span, we observed a 30% PR rate, with a median PFS of 4 months with two chemotherapy regimens commonly used in other soft tissue sarcomas. In our series, the patients were relatively young, and the retrospectivity of the analysis makes it hard to transpose data on toxicity of these two regimens to the general population.

The benefit of multi-agent compared to single agent anthracycline-based first line chemotherapy for advanced soft tissue sarcomas remains controversial. In the biggest randomized prospective trial, the combination of doxorubicin and ifosfamide was associated with higher response rates, but a non-significant overall survival benefit [24]. However, upfront combination chemotherapy may be of benefit, even considering the increased toxicity, for selected patients, e.g. those with a high tumor burden requiring a prompt tumor shrinkage or those with a fast-growing disease, in need of obtaining a response. In terms of responses to anthracycline and ifosfamide, our results point to an antitumor activity similar to advanced soft tissue sarcomas in general [24]. However, PFS was markedly low in our series. The limited number of patients makes it difficult to speculate on this.

HDIFX is often used as a salvage regimen in synovial sarcoma and dedifferentiated liposarcoma, even in patients previously treated with standard-dose ifosfamide [25–29]. Furthermore, if compared with the classic ifosfamide schedule, the administration in the external portable device in a prolonged 14-days continuous infusion shows an excellent tolerability. Main toxicities are represented by mild to moderate nausea and vomiting, while myelosuppression, neurotoxicity and acute kidney failure are infrequent.

In our series, the anti-tumor activity of HDIFX as second/further chemotherapy line in myxofibrosarcoma was apparently higher than in other soft tissue sarcomas [26–30].

Among 28 patients treated with HDIFX in second/further line, the PR rate was 36% and median PFS was 4 months. We reported a similar PR rate for continuous-infusion HDIFX in well differentiated/dedifferentiated liposarcomas [25]. Nine patients were pretreated with first-line epirubicin–ifosfamide and we observed 4 PR (31% RECIST PR) out of 13 patients evaluable for response. Thus, high-dose ifosfamide was still active in patients pre-treated with anthracyclines and standard-dose ifosfamide. Again, however, median PFS was low. The previous exposure to ifosfamide may, in part, explain these results.

In advanced soft tissue sarcomas, standard chemotherapy is based on anthracyclines as the first-line treatment and the sequencing of second-line, third-line and fourth-line treatments remains open to debate [13, 31, 32]. Histological diagnosis can help guide the sequence of treatments [32]. Indeed, a histology-driven choice is feasible in some histologies (e.g., paclitaxel for angiosarcoma, gemcitabine for leiomyosarcoma, trabectedin for myxoid liposarcoma, etc.). In this perspective, the reasonably interesting PR rate seen with continuous-infusion HDIFX may be of interest.

## Conclusions

This was a retrospective analysis in a relatively young small population of patients over a 15-year span. However, among soft tissue sarcomas, myxofibrosarcoma represents a rare subtype. No data are reported so far on the activity of chemotherapy and no prospective neither retrospective studies focusing on its medical treatment are available. Obviously, it is difficult to conceive prospective studies in such a rare subtype. In this sense, this report adds some information which may assist the clinicians in the choice of regimens. However, the low PFS implies that we are in need for new therapies for such a histology, when a medical treatment is required.
